# Patients’ Attitudes Toward Electronic Health Information Exchange: Qualitative Study

**DOI:** 10.2196/jmir.1164

**Published:** 2009-08-06

**Authors:** Steven R Simon, J Stewart Evans, Alison Benjamin, David Delano, David W Bates

**Affiliations:** ^5^Department of Clinical and Quality AnalysisPartners HealthCare SystemIncBostonMA; ^4^Division of General Internal MedicineBrigham and Women's HospitalBostonMA; ^3^Northern Berkshire eHealth CollaborativeNorth AdamsMA; ^2^Division of Clinical Decision MakingInformatics and TelemedicineDepartment of MedicineTufts-New England Medical CenterTufts University School of MedicineBostonMA; ^1^Department of Population MedicineHarvard Medical School and Harvard Pilgrim Health CareBostonMA

**Keywords:** Regional health information organizations, health information technology, electronic health records, quality of health care

## Abstract

**Background:**

In many countries, there has been substantial progress in establishing the electronic transmission of patients’ health information between health care providers, but little is known about how best to engage patients in the process.

**Objective:**

We explored patients’ views about sharing of electronic health information and their preferences for learning about and participating in this process.

**Methods:**

Patients in one Massachusetts community in the northeastern United States were recruited to participate in focus-group discussions. Prior to discussion, participants completed a written questionnaire that captured their reactions to draft educational materials and a consent form. The discussion moderator and two physicians analyzed the moderator’s detailed notes from each session and participants’ written comments, using an immersion-crystallization approach.

**Results:**

Three dominant themes emerged: (1) concerns about privacy and security, (2) the potential benefit to a person’s health, and (3) the desire for more information about the consent process. On the pre-discussion questionnaire, 55 out of 62 participants (88%) indicated that they would provide consent for their information to be shared electronically among their health care providers, given the materials they had reviewed.

**Conclusions:**

Patients are enthusiastic about electronic health information exchange, recognizing its capacity to improve the quality and safety of health care; however, they are also concerned about its potential to result in breached privacy and misuse of health data. As the exchange of electronic health information becomes more widespread, policy makers will need to ensure that patients have access to concise educational materials and opportunities to engage in conversations about the risks and benefits of participation.

## Introduction

The United States has lagged behind other developed nations with respect to adoption of electronic health records (EHRs), especially in primary care, although that appears likely to change. The American Recovery and Reinvestment Act of 2009 will result in an investment of approximately US $19 billion toward the adoption of EHRs and, under the direction of the Office of the National Coordinator for Health Information Technology, an “initial set of standards, implementation specifications, and certification criteria” to enable the electronic exchange of health information [1]. With these developments in the United States, electronic sharing of health information—typically defined as the exchange of personal health information contained in the medical record between at least two different computer networks—is expected to increase exponentially over the next decade [2]. The expansion of electronic data sharing, also known as health information exchange (HIE), is heralded as a key solution to the problems of low quality and high cost of health care [3]. However, HIE has presented substantial challenges, not only in the United States but also in countries such as the United Kingdom [4], Australia [5], and Sweden [6].

The potential benefits of electronic health information exchange include improved health care quality, reduced medical errors and lower health care costs, as well as public health benefits, resulting from early detection of infectious disease and improved tracking of chronic disease management [7]. In the United States, regional health information organizations (RHIOs) have emerged as the leading model to facilitate the electronic exchange of patient-level clinical information between physicians’ offices and other health-care organizations that deliver care to patients [8,9]. The overarching goal of these RHIOs is to ensure that physicians and other health care providers have access to the best and most complete information about patients for whom they are caring when they need it most—in real time, when the patient is with them in the examination room, emergency department, or other clinical setting. Not only could awareness of a patient’s medical history, such as, for example, a current problem list or known allergies to medications, improve the quality of health care, but it could be, in some instances, life-saving [10]. The model of RHIOs is considerably different from that being used in other nations such as the United Kingdom, which has a single “spine” which is being implemented [11].

To date in the United States, however, the electronic exchange of health information has faced considerable challenges with respect to technical limitations and financial constraints (e.g., is there a sustainable business model?) [12]. Overcoming the lack of interoperability (i.e., the problem of systems on different platforms being unable to exchange information) and the question of who will pay for health information exchange have presented major impediments. In addition, concerns about the privacy and security of personal health information in these systems have also emerged [13,14]. As communities begin to overcome the technical and financial barriers to health information exchange, attention has turned to the importance of engaging community members—the patients—in the process. Specifically, policymakers have begun to focus on patients’ perspectives of electronic health information exchange, especially their concerns about confidentiality and data security, and their willingness to provide consent for health information exchange.

In preparation for launching community-wide electronic health information exchange in Massachusetts, we conducted formal and informal discussions among stakeholders to address key questions about the process: How well do patients understand the value of clinical data exchange? To what extent do they endorse the electronic transmission of clinical information among health care providers? What are their concerns and hesitations about the process? How should patients be informed about, and approached for, participation in a community’s HIE?

## Methods

### Study Design

We conducted a qualitative analysis based on transcribed moderators’ notes from five focus group discussions and on the free-text and responses from semi-structured questionnaires completed anonymously by focus group discussion participants. The research protocol was approved by the Partners HealthCare Human Research Committee.

### Setting

The study was conducted in the Northern Berkshire e-Health Collaborative, one of three communities participating in the Massachusetts e-Health Collaborative. The Northern Berkshire community is a rural, socio-economically diverse region with a population of about 45,000 people, located in the northwestern corner of Massachusetts. It includes the city of North Adams and several smaller towns that surround it. Northern Berkshire community members receive the majority of their health care services from physicians and other health care professionals located in North Adams, Adams, and Williamstown. Focus group discussions were conducted at various locations, including community centers, health care facilities, and restaurants, to solicit a broad spectrum of patients’ attitudes and perceptions.

### Participants

We recruited adult community members to participate in focus group discussions based on their geographic proximity and affiliation with the site at which each session was being held. For example, for a session conducted at the North Adams Regional Hospital, we recruited hospital employees. We advertised the focus groups at each location, with posted signs, flyers, and email announcements used to recruit participants. We aimed to include a mix of men and women, ranging from young adults to senior citizens, and attempted to include individuals with varying levels of formal education.

### Focus Group Discussions

A trained moderator conducted each of the focus group discussions, with one or two additional observers present for each session. The duration of each session ranged from 30 to 120 minutes. The sessions were not audio recorded; however, the moderator and observers took extensive notes and recorded their collective observations from discussions that followed the sessions.

At the start of each session, prior to any discussion, we asked participants to review the following draft materials as though they were seeing them for the first time at a routine visit to their doctor’s office:

- Form seeking consent for patients to allow their doctor’s office to share health information with other physicians’ offices. (See Multimedia Appendix 1)
- Booklet of supporting information, describing the health information exchange, data security, privacy considerations, and contact information. (See Multimedia Appendix 2)

After reading the materials, participants were asked to complete a one-page questionnaire that ascertained their age, sex, highest education level attained, and health condition (overall healthy, healthy now but with past concerns, some current health concerns, significant current health concerns). In addition, the questionnaire asked whether they would sign the consent form that they had just read.

During each session, the moderator facilitated discussion of individuals’ reactions to the documents, including their likes and dislikes, preferences for additional information, reservations about the forms or the HIE itself, and thoughts on improving the documents. Participants were encouraged to ask questions about anything that seemed unclear. Because of scheduling issues, one of the five sessions was conducted as a series of one-on-one interviews between the moderator and individual community members as they arrived at the planned focus group meeting. The content of these interviews was similar to the group discussions and was included without discrimination in this analysis.

### Analysis

We performed a content analysis of the moderators’ notes and free-text comments from the questionnaires using an immersion-crystallization technique [15]. Three of us (SRS, JSE, AB) read all of the available text and identified the salient themes and principles that emerged. Any discrepancies were settled by consensus. The raw rate of willingness to provide consent for HIE was calculated from the survey forms and stratified by age group, gender, education level, and health condition (healthy vs current health concerns).

## Results

### Participant Characteristics

Of the 64 study participants, 61 (95%) provided information on their age, sex, and health status. The median age was 50 years (range: 19 to 86 years), and 46 patients (75%) were women. A total of 52 participants (85%) had attended college or some graduate school, reflecting a sample with higher education level than the community at-large. A total of 36 patients (59%) considered themselves generally healthy, while 17 (28%) said that they had some current health concerns.

### Salient Themes

The three most common themes that emerged from the focus group discussions and qualitative comments from the written questionnaires were (1) concerns about privacy and security, (2) the potential benefit to a person’s health, and (3) the desire for more information about the consent process.

#### Privacy and Security Concerns

Comments and discussion about privacy ranged from general concerns about privacy to specific concerns about who will have access to the personal health information, what kinds of sensitive health information would be shared, and the risk of unauthorized access to the health information via security breaches. One woman who was “over 65”, expressed her acceptance of health professionals' sharing her data but her simultaneous reservation about unauthorized access:

I realize that people in the office already can look at my chart. But I’m worried about people that don’t need to know—hackers on the outside. What they would do with the information, I don’t know, but I still don’t like it.

Others expressed a considerable level of trust in the security of the system. For example, participants were generally nonchalant when informed that some potentially sensitive health information could be shared among physicians’ offices. When the moderator noted, for example, that prescriptions for mental health conditions or for erectile dysfunction would be viewable by multiple providers, there was no measurable pushback. One man commented:

Yeah, but the doctors [already] ask you about all that stuff anyway, right? This isn’t really that different.

#### Potential Health Benefits

Across a wide spectrum of participants, the potential for health information exchange to improve health and prevent adverse outcomes was unambiguously endorsed as a rationale for participating. The health benefits of electronic health information exchange were cited with equal frequency among those who had concerns about privacy and security as those who had no reservations about proceeding with the community-wide clinical data exchange. For example, one man with no concerns about security proclaimed:

Yeah, I'd sign that [consent form]. It's for my benefit so why not? Nobody can get at the information, so what the heck difference does it make anyway?

A 29-year-old woman who has some chronic medical conditions said that health information exchange was a “great idea” and would “make the process of seeing a specialist much easier”. Another woman, who mentioned that her father had a chronic illness, reported that his paper medical chart had recently been lost at the doctor’s office and expressed optimism that an electronic record with health information exchange would avoid that problem:

It was extremely important, and we just don’t know what happened to it. If this prevents that from happening, it’s a good thing.

#### Desire for More Information

Some participants expressed high levels of satisfaction with the draft document presented during the focus groups and requested no further explanation or information. In fact, at a focus group held at a local restaurant, several middle-aged men completed the draft forms and were initially under the impression that they were signing the official documents. Nevertheless, participants throughout all demographic groups suggested that the process of obtaining patient consent for opting in to the system of health information exchange would require considerable patience and more extensive information resources. Virtually all patients expressed the sentiment that they should need to provide consent for health information exchange (i.e., an opt-in system); a system that assumed their willingness to participate without obtaining explicit consent (i.e., an opt-out system) would not be acceptable.

There was consensus in all groups that patients should receive information by mail prior to being asked to sign the consent form in the doctor’s office. While there were a few individuals who said that they would simply sign the consent form without reading it carefully, most participants said that they would want to take time to read it thoroughly to consider whether to participate. Focus group participants suggested that patients could be warned to arrive 10 - 15 minutes prior to their appointment to enable time for the registration and consent process to occur. Patients agreed that someone in the physician’s office needs to be dedicated to the sign-up/consent process:

If you send an advance notice, you’ll need to be prepared for a lot of calls. You’ll need someone who can take the time to answer people’s questions, both when the mailing goes out, and when people come in the office.

One older woman who described herself as ambivalent about whether to opt in to the HIE noted that both of her physicians were included on the list of participating clinicians and then commented:

I would have to discuss it very thoroughly with them—what type of personal information they’re putting in there.

### Willingness to Provide Consent

In completing the written questionnaires prior to beginning the discussion component of the focus group meetings, 55 out of 62 participants (88%) indicated that they would provide consent for participation in the system of health information exchange, given the materials they had reviewed. While the study was not designed to detect differences in the rates of consent among demographic subgroups, the proportion of participants willing to opt in was qualitatively similar among men and women, younger and older individuals, and those with and without current health concerns, and it was not related to the level of education.

## Discussion

Exchanging health-related information electronically to improve clinical practice is central to maximizing the benefit of ongoing efforts to expand electronic health records to physicians’ offices everywhere [16]. Because health information exchange involves electronically exchanging patient-identified health information across geographically and commercially separate entities, it raises issues of patient privacy and data security which have resulted in heated debate [17,18]. However, little is known about patients’ attitudes toward health information exchange and their preferences for learning about it and giving consent for it. In this qualitative analysis of five focus groups within a community on the eve of launching a regional system of health information exchange, we found that most patients were willing to opt in to a system of health information exchange, although they had concerns about security and privacy and wanted assurances that they would be able to ask questions and obtain more information prior to consenting. Overwhelmingly, patients recognized the potential for the electronic exchange of health information to improve the quality of health care and prevent medical errors. This potential was the driving force behind many patients’ enthusiasm for participation.

Patients’ concerns about privacy and data security are not surprising, given the attention paid to these issues in both the medical and lay literature [19,20]. Furthermore, there have been some well-publicized breaches, such as an incident in the United Kingdom in which data from 25 million individuals were placed on CDs that were lost in the mail [21]. While some have pointed out that paper-based records systems have long been subject to breaches of privacy and security, the potential for large volumes of electronic data to be accessed in short amounts of time and the ease with which those data can be transmitted from user to user have elevated concerns about potential privacy violations and security breaches in computerized systems [22]. In addition, the high visibility of news stories which depict data loss and security breaches in a variety of business sectors [23], as well as in health care [24,25], seems to have sensitized the public to these concerns in the context of health information exchange. As countries intensify health information exchange efforts, concerns about privacy and data security are likely to increase, as well. For example, in the United Kingdom, where the National Health Service transmits more than 100 million clinical messages electronically every month [26], public concern has been particularly vocal and substantially hampered efforts to advance clinical information exchange programs [27,28]. It is reasonable to expect that public concern in other countries will increase correspondingly as the volume of data exchange expands to these levels.

In the United States, most early data exchange efforts have been at the community level. In this study, the concerns about privacy and security ranged from non-specific, “gut-level” worries to sophisticated, reasoned acknowledgements of the risks in both electronic and paper-based systems. Without question, though, patients across the community in our study expected privacy and security to be addressed and adequately assured.

Among community members, there was unanimous acceptance of the notion that health information exchange would lead to improvements in the quality and safety of health care. Patients cited their own experiences with misplaced paper charts, for example. They easily recognized the potential value of having their health information, such as medication lists or allergies, immediately accessible to clinicians in practice settings where they are not customarily available. In this study, no one expressed any skepticism about the potential for health information exchange to improve care, and in fact many patients linked this potential benefit to their willingness to provide consent for participation. The promise of improved quality and safety of health care seemed to outweigh patients’ deep-seated concerns about confidentiality and data security, as long as the system explicitly and adequately addressed the latter.

Participants in this study also articulated a consistent message that community members must receive clear and concise materials describing the system of health information exchange and have opportunities to ask questions about it before they would be willing to opt in. While nearly all participants indicated that they would provide consent for inclusion in the exchange system, many expressed a need to have information about it, and especially assurances of privacy and security, available for review and consideration well in advance of being asked to “sign on the dotted line”. This finding indicates that enrolling a community in health information exchange may take considerable time, as patients want to be able to read and reflect on materials, discuss them with family members, and ask clarifying questions before committing to it. Patients’ interest in making an informed decision also likely reflects the fact that they expect to have control over whether their information is included in the system.

The input from the focus group discussions led to considerable revision of the consent form (See Multimedia Appendix 3), the educational / informational brochure (See Multimedia Appendix 4), and the fact sheet (See Multimedia Appendix 5) that were ultimately produced and distributed within the community. In the initial materials and during the discussions, for example, the health information exchange was referred to as a community health record or a shared health record. Focus group discussions suggested that “e-health summary” was a more appealing and meaningful description. In addition, feedback from patients led program leaders to craft a schematic diagram of the multiple sources of information that would populate the e-health summary (See Multimedia Appendix 4, page 5).

Perhaps the most noteworthy outcome of the focus group discussions was the decision to make health information exchange an “opt-in”, rather than and “opt-out”, experience. At the time of the focus group discussions, regional policymakers had considered establishing the health information exchange system such that the records of all patients of all participating physicians would be included unless the patient actively opted out. However, patients’ robust preferences for retaining the authority to provide consent—and their near unanimous expressions of willingness to provide that consent—led to the establishment of an “opt-in” system.

A few prior studies from the United States have explored patients’ attitudes toward the use and protection of health information [29-31]. Little is known about how best to approach the process of engaging community members in health information exchange in the United States and abroad. One of the first regional health information exchange systems in the United States, the Santa Barbara County Care Data Exchange, recognized that privacy issues need to be addressed explicitly and early in the process [32]. Santa Barbara leaders also realized the value of engaging community members in the development of policies intended to educate consenting patients before they participated in the health information exchange [33]. These observations are consistent with and reinforced by the salient themes that emerged from our study.

Interestingly, some experts have suggested the potential for streamlining the consent process for health information exchange through electronic communication [34]. While our study did not directly assess the acceptability of an e-consent process, we did find that community members expect information to be presented clearly and concisely, with time provided between the presentation of information and the need to consent in order for there to be opportunities to ask for more or clarified information. To the extent that electronic consent processes can incorporate these community needs, they may enable communities to streamline the enrollment process, though access to paper materials and human information sources will likely remain essential.

The findings of this analysis need to be considered in the context of the study design. We conducted five focus groups in one community in Massachusetts, and the attitudes and preferences of patients in communities elsewhere may differ. On the other hand, the purpose of this study was to identify the breadth of attitudes among community members about consent for health information exchange and, as such, it is likely to have identified the relevant domains of concern for many other communities embarking on similar efforts.

This study provides insight into the ways that patients perceive electronic health information exchange and their willingness to provide consent for participation. Others have already recognized the value of sharing experiences and lessons learned from community implementation of health information technology, including the electronic exchange of health information [9,35]. Future studies should test differing strategies for educating community members about health information exchange and for securing their consent for participation. While there will likely be variability across communities and nations, as well as a need for local programs and policies, each community embarking on the implementation of clinical data exchange should not need to “reinvent the wheel” in terms of engaging patients in the process.

## Multimedia Appendix 1

Draft informed consent form

## Multimedia Appendix 2

Booklet of supporting information

## Multimedia Appendix 3

Patient consent form to allow sharing of medical information via the health information exchange

## Multimedia Appendix 4

Educational / informational brochure describing electronic health records and the health information exchange

## Multimedia Appendix 5

Information sheet mailed in advance to members of the Northern Berkshire Community

## Abbreviations

EHR: electronic health records
HIE: health information exchange
RHIO: regional health information organizations
