# Supplementary material for: Patients’ Attitudes Toward Electronic Health Information Exchange: Qualitative Study
Source: J Med Internet Res. 2009 Aug 6;11(3):e30. doi: 10.2196/jmir.1164 (PMC2762851; doi:10.2196/jmir.1164)
Supplement: Supplementary file 1 [file jmir_v11i3e30_app1.pdf]

Appendix 1. Draft informed consent form. This two-page form was shown to focus group participants prior to any discussion and prior to their completion of a questionnaire assessing their willingness to opt-in to the health information exchange.

Permission to Share My Medical Information with Other Health Care Providers Using an Electronic Community Record

Your physician requests your permission to share your medical information with authorized providers in the community who are involved with your care, using a secure electronic format known as the Electronic Community Record. The Electronic Community Record will contain a single, summarized view of relevant parts of your medical record. It will not contain the office notes your provider creates on your behalf.

The information to be shared by participating health care providers include the same categories of information already being shared today via other methods of communication.

This new method of sharing information will enable participating clinicians who are involved with your care to access your vital medical information more comprehensively and quickly than current, paper-based methods. Any health care provider authorized to access this information has agreed to implement and abide by specific monitoring and security principles aimed at protecting patient data. Since this is a new program, we are requesting your specific permission to share your health information through this Electronic Community Record for the purposes described above, including, but not limited to, classes of information identified as “sensitive” according to Massachusetts Law (for a discussion of various categories of sensitive information that may be included in the Electronic Community Record under this consent, see the attached brochure). HIV/AIDS diagnoses and genetic testing will <u>not</u> be included in the Electronic Community Record without your additional specific consent.

This Electronic Community Record will be accessible only as needed for your care, and only by authorized practices who have agreed to work collaboratively in the best interest of their patients and to abide by certain rules for accessing your Record. Current participants include *{21 entities were listed here, including medical practices, hospitals, and other health care facilities}*. This list may change over time, as new practices are added and deleted, and at any time, you may request a list of current participants from your doctor’s office.

By my signature below, I acknowledge that I have been provided with sufficient information and have had an opportunity to have my questions answered about the Electronic Community Record. I hereby grant permission to the above organizations to use, and disclose my information to other participating health care providers and health care organizations with similar access to the Electronic Community Record in connection

DRAFT

with my care. I understand that by refusing to sign this form, my medical information will not be immediately accessible through an Electronic Community Record, which means it will not be available at other participating providers' offices, including the emergency room. I further understand that I may opt-out of the Electronic Community Record at any time with written notification. Should I choose to opt-out at a later point, the opt-out will be effective within 1 business day of providing this written notification, but any information provided prior to that date will remain in the Electronic Community Record.

________________________________ ____________________________

Signature of Patient/Representative Date

DRAFT
