# Supplementary material for: Patients’ Attitudes Toward Electronic Health Information Exchange: Qualitative Study
Source: J Med Internet Res. 2009 Aug 6;11(3):e30. doi: 10.2196/jmir.1164 (PMC2762851; doi:10.2196/jmir.1164)
Supplement: Supplementary file 2 [file jmir_v11i3e30_app2.pdf]

**Appendix 2. Booklet of supporting information. This form was shown to focus group participants prior to any discussion and prior to their completion of a questionnaire assessing their willingness to opt-in to the health information exchange.**

# Supporting information:

To be provided in booklet form. Booklet will likely be 12 pages, larger type, 4-color, with many graphics. Will be reviewed/edited by a medical literacy expert prior to printing.

We may provide a 1 page “Quick Start” summary – NOT included in this packet. This 1-pager will provide a fast, easy-to-read overview of the project, the electronic health record vs. the ECR, and the implications of signing (or not signing) the consent form.

# Frequently Asked Questions about your electronic medical record

Your doctor requires your permission to share your medical information with other community health care providers (doctors, nurses) who are involved with your care. This information sharing will be done via technology called the "Electronic Community Record." The Electronic Community Record is a secure method for information-sharing among providers – one which can be tracked and audited for your security. This brochure answers some of the most common questions about the Electronic Community Record, and about the privacy and security of your record in general.

***What exactly is the Electronic Community Record?***
The Electronic Community Record contains information about your health that has been identified as important for any of your doctors to know. Because the information is centralized and updated on an ongoing basis, it allows any doctor that treats you within the community who has agreed to participate in the overall Electronic Community Record project to have the most up-to-date information about your health – whenever and wherever he or she needs it for your medical care.

***Why is it important that my information be shared between providers?***
Your doctor strongly recommends that you permit sharing of your medical record, because doing so can help improve the quality and safety of your medical care. For example, in the case of a medical emergency, the doctor treating you will have a list of your current medications, allergies, and other health conditions. This could potentially save your life. In a non-emergency situation, it will allow for more effective care, and reduce redundancies (i.e. having different doctors recommend getting the same lab test). Finally, it will allow you to spend better quality time with your physician, helping improve your care.

***Are electronic records as secure and private as paper records?***
If managed appropriately, they are more secure. Your electronic record, unlike a paper record, tracks exactly who has viewed or changed a record, what information was accessed, and when. It allows multiple levels of access, so physicians can have access to confidential information that other staff members cannot. It automatically "blocks" diagnoses protected by law (HIV, genetic testing) from any public view, unless you provide additional permission for access. It is guarded by technology as good as, and in some cases better than, the systems that world-leading banks use to protect financial data. These protections include password protection, firewalls, intrusion detection, data encryption, and secure SSL technology. Finally, your medical information will remain more secure than a paper record if a fire, natural disaster, or other event should occur. For more information on the security of your paper record, please see **Security of Your Health Information**.

***What about office staff? Will everyone in a practice be able to view my medical information?***

No. Because multiple levels of access are allowed, different levels of information will be accessible, depending on who is viewing your record. For example, in most instances, the front desk staff at a practice will view only demographic information (name, address, insurance carrier, etc.). The nurse or doctor treating you will be able view more detailed health information like diagnoses, medications, and past medical history, and recent medical tests.

***Why would my different doctors need to share information, when I'm seeing them for totally different reasons?***
Because your body works as a system, health issues that seem unrelated often have significant – and potentially dangerous – clinical overlap. For example common heart medications can have dangerous interactions with over-the-counter pain medications. Other heart medications are actually fatal with common prescriptions like Viagra. Antibiotics can reduce the effectiveness of oral contraceptives. Patients with heart disease or diabetes are often at increased risk for other conditions, like depression. These are just a few examples – there are literally tens of thousands of such examples. That's why it is important that all doctors treating you have access the full spectrum of information.

***What if there's some specific, personal information that I want to keep private?***
The Electronic Community Record will contain those categories of information determined by doctors to be most important for your safety and quality care. Consistent with Massachusetts law, no health information will be contained in the Electronic Community Record without your consent, and information about HIV/AIDS or genetic testing will be added to the Electronic Community Record only with your additional and specific consent. Please be aware that the Electronic Community Record may contain categories of information that are considered 'sensitive' under Massachusetts General Law (including mental health, infectious & venereal disease, abortion, child abuse, domestic abuse/sexual assault, mammography, research involving controlled substances and substance abuse). This is the same information that is currently being shared using other methods as needed for appropriate medical care. In addition, your doctor will have the ability to keep information confidential by placing it in a secure, private place within your records. You should be sure to discuss with your doctor any information that you want to remain private.

***How can I be sure that my records are accurate, and that they are being viewed and used appropriately?***
Just as with paper records, you are entitled to a copy of your medical record at any time, for any reason. With electronic medical records, you may also request at any time a record of who has accessed your medical information, when they accessed it and from where they accessed it.  This is an improvement in the security of your information from the current paper-based records.

***What happens if my health information is disclosed to or accessed by someone not involved with my care?***
Those participating the Electronic Community Record project have taken reasonable and appropriate steps to protect your health information. Those who are managing the

Electronic Community Record have built “alarms” to identify potential problems and perform regular audits to check for proper use of the system and to identify potential security concerns.  Federal laws and state laws make inappropriate access to your health information a crime that is punishable with fines as much as $250,000 and 10-years imprisonment.  If it is determined that your information has been disclosed to or obtained by someone not authorized, the managers of the Electronic Community Record will be responsible for investigating and resolving the situation, including taking steps to press charges to the full extent of the law.  In the case of employees of participants in the Electronic Community Record, unauthorized disclosure or access can also result in swift termination of employment by the respective participant.

***Will I still need to fill out the same medical forms everywhere I go?***
If you sign the Electronic Community Record consent form, when you arrive at a new doctor's office within the community, you will be able to spend your time on quality care, instead of answering the same questions you have already told another provider. While your doctor may want to confirm the information, or make sure there have been no changes, this process will likely be much more efficient. If you see a physician outside of this community, however, you may need to provide this information again.

***Why are you asking me to sign a form?***
We need your permission to create an Electronic Community Record for you. If you do not sign the consent form, your information will not be made available electronically to other providers, including the emergency room.

***How can you give me comfort that that having my information available through the Electronic Community Record is in my best interests?***
This technology has been endorsed by….<<<MENTION SOME GOOD CONSUMER/ SAFETY GROUPS HERE>>> (endorsements currently on the way).

# Security of Your Medical Record

The Northern Berkshire eHealth Community is comprised of many different health care providers who have agreed to work collaboratively in the best interest of our patients. At present, participants include *{21 entities were listed here, including medical practices, hospitals, and other health care facilities}.* This list may change over time, as new practices are added and deleted, and at any time, you may request a list of current participants from your doctor's office.

Every practice in the Northern Berkshire eHealth Community is fully committed to the privacy, security, and confidentiality of your medical record. Your trust is important to us, and with changes in technology, protecting your patient records remains a foremost priority. In protecting your record, we use secure technology, and we provide rigorous, ongoing training regarding the confidentiality and security of health records.

There are two levels of your electronic health record, each with security features:

- Your **electronic health record** is not optional; this is how your medical provider now keeps your records. Your record will be kept within the practice only, unless you have expressly given your consent to share certain components of your medical record via the "electronic community record."

- The **electronic community record** is a secure, private record that can be shared between authorized providers at different health care practices within the community. You must give your consent before this information can be exchanged between and among these health care providers. The electronic community record contains a collective "snapshot" of your most important health concerns, including those considered "sensitive" under applicable Massachusetts law (for a discussion of various categories of sensitive information that may be included in the electronic community record under this consent, see the attached brochure). HIV/AIDS diagnoses and genetic testing will <u>not</u> be included in the electronic community record without your additional specific consent.

Both your electronic health record and your electronic community record are secured by rigorous technologies that represent a significant improvement over paper records: firewalls, intrusion detection, secure (SSL) communications, password protection, automatic alarms for suspicious activity, and role-based access (so that non-clinical staff can be "blocked" from viewing medical information). In addition, the electronic community record is protected by data encryption, so that data appears scrambled to a non-authorized user. Both the electronic health record and electronic community record track who has viewed or changed a record, and what information was accessed /altered, using a date/time stamp for each access point.

At any time, you may request an audit trail of who has viewed your record, and when. Our community will also be performing regular audits to ensure that no one has improperly accessed your record. Each health care provider will be able to supply you with a notice of its privacy practices.

We value our relationship with you, and we are committed to protecting your medical information.

Electronic Health Records vs. Paper Records
A Comparison of Security Attributes

| | Paper | Electronic |
|---|---|---|
| Patient has right to request copy of medical record at any time, for any reason | Yes | Yes |
| Practice provides regular training to staff on maintaining privacy and security | Yes | Yes |
| Tracks who looked at record, what section of record they viewed, and date/time of viewing | No | Yes |
| Monitors whenever information is added, deleted, or edited, along with the name of the individual who made the change, and date/time of change | No | Yes |
| Can easily provide patient with an audit trail of who has looked at record and when | No | Yes |
| Protected by firewalls, intrusion detection, and secure SSL technology | No | Yes |
| Password-protected | No | Yes |
| Option to protect with biometrics, other | No | Yes |
| Allows different levels of access, depending on sensitivity of information (I.e. physicians can have access to more personal information) | No | Yes |
| Information complete | No | Yes |
| Information in the right place at the right time | No | Yes |
| Can usually be recovered in case of fire, natural disaster, or other major event | No | Yes |
| Generates automatic "alarms" about any suspicious use | No | Yes |
| Ability to build in additional rules about who accesses charts | No | Yes |
| Violations punishable by law | Yes | Yes |
| Information shared with employers | No | No |
| Information 'sold' to other parties | No | No |
| | | |
| | | |
