# Supplementary material for: Patients’ Attitudes Toward Electronic Health Information Exchange: Qualitative Study
Source: J Med Internet Res. 2009 Aug 6;11(3):e30. doi: 10.2196/jmir.1164 (PMC2762851; doi:10.2196/jmir.1164)
Supplement: Supplementary file 3 [file jmir_v11i3e30_app3.pdf]

Appendix 3. Patient consent form to allow sharing of medical information via the health information exchange (HIE).

— Permission to Create an *eHealth Summary* and Share My Medical Information —

We are taking part in an exciting program to improve your health care and make office visits easier and more convenient. To do this, your doctor would like your permission to enroll you in our *eHealth Summary* program. This means sharing important parts of your medical information with other providers (doctors, nurses and health professionals) through an electronic medical chart. Only authorized healthcare professionals, their agents and others whose job it is to secure, monitor and evaluate the operation of the information system and quality of care would be able to access your information. The *eHealth Summary* will allow your providers to access your health information more quickly and accurately than with paper charts.

The *eHealth Summary* is an overview of vital medical information. For instance, the eHealth Summary may include a list of your current medications, allergies, recent diagnoses (problems) and any surgery you may have had. It will not include detailed confidential notes from your office visits. Information in the *eHealth Summary* may include, but is not limited to, that which Massachusetts law considers "sensitive" such as mental health, substance abuse, sexually transmitted disease, and sexual abuse information. HIV/AIDS diagnoses and any genetic testing results for health screening purposes will not be included in the *eHealth Summary* without your written permission each time it is used.

The *eHealth Summary* has a secure system to protect your healthcare information. All authorized healthcare professionals with access to the *eHealth Summary* agree to follow strict privacy and security policies. Technology will encrypt (scramble) the information and track who and when someone has accessed your summary. You may request a list from your doctor's office of who has accessed your electronic records.

Your doctor is asking permission to share your vital medical information through the eHealth Summary for all legally permitted uses and disclosures. These include but are not limited to:

- Clinical care • Billing and financial management • Administrative management
- Reports to public health agencies and other governmental requirements
- Reports to protect the security of your medical information
- Reports to evaluate the use of the *eHealth Summary*
- Reports to track and evaluate the quality of your healthcare services

Privacy risks include the possibility that your medical history could indicate specific conditions such as sexually transmitted diseases, mental health, pregnancy, HIV status, genetic conditions, chronic conditions and alcohol or drug problems, or other conditions that you may consider sensitive. Other risks may include unauthorized individuals gaining access to the computer network, an authorized user misusing the health information and an authorized person seeing information in your record that makes you uncomfortable.

There is also the possibility of inaccurate information being accidentally entered into your electronic record and shared with others through your eHealth Summary which could have a negative impact on your care.

You can learn more about the *eHealth Summary* by reading the information booklet, "Understanding Electronic Health Records" that your doctor's office has provided.

❑ **Yes, I want my health information included in the Northern Berkshire eHealth Collaborative *eHealth Summary* as described above and in the provided information booklet. By my signature below:**

I acknowledge that I have been given sufficient information and have had the opportunity to have my questions answered about the *eHealth Summary.*

I give permission to those described above to use and disclose my information, as described above and in the provided information booklet.

I understand that I have the option to withdraw permission and can do so by giving written notice to my doctor's office. Should I withdraw my permission, this request will be effective within one (1) business day of my written notice.

____ ______________

Signature of Patient/Representative *Date*

❑ **No, I do not want my information included in the Northern Berkshire eHealth Collaborative *eHealth Summary*.**

I understand that my information will still be stored electronically for my provider's records, but an *eHealth Summary* will not be available to other providers (including the North Adams Regional Hospital). I also understand that, without the *eHealth Summary*, it may be more difficult for doctors and healthcare providers to coordinate my care. This could have an adverse effect on the quality and efficiency of my health care services.

____ ______________

Signature of Patient/Representative *Date*

The following providers will have access to the eHealth Summary:

- Adams Internists
- Associates in Urology
- Berkshire Hematology Oncology
- Greylock Gastroenterology
- Herr, Douglas V, MD, Berkshire Cardiology
- Hertzig, Gerrity, Griffin, & Degrenier
- Howland, John MD
- Integrative Medicine
- Maher, Paul MD
- North Adams Regional Hospital
- Northern Berkshire Family Practice
- Northern Berkshire Sports Medicine
- Orthopedic Associates of Northern Berkshire
- Sweet Brook Transitional Care and Living Centers
- Tanzman, Maselli, and Associates
- Van Uitert, Robert L. MD
- VNA + Hospice of Northern Berkshire
- Williamstown Medical Associates
- Yurfest, Joshua T. M.D.

This list may change as new providers are added and removed. You may request a list of current providers from your doctor's office.
