# Supplementary material for: Patients’ Attitudes Toward Electronic Health Information Exchange: Qualitative Study
Source: J Med Internet Res. 2009 Aug 6;11(3):e30. doi: 10.2196/jmir.1164 (PMC2762851; doi:10.2196/jmir.1164)
Supplement: Supplementary file 4 [file jmir_v11i3e30_app4.pdf]

Appendix 4. Educational/informational brochure describing electronic health records (EHRs)and the health information exchange (HIE).

NBeHC

Northern Berkshire eHealth Collaborative

Understanding Electronic Health Records

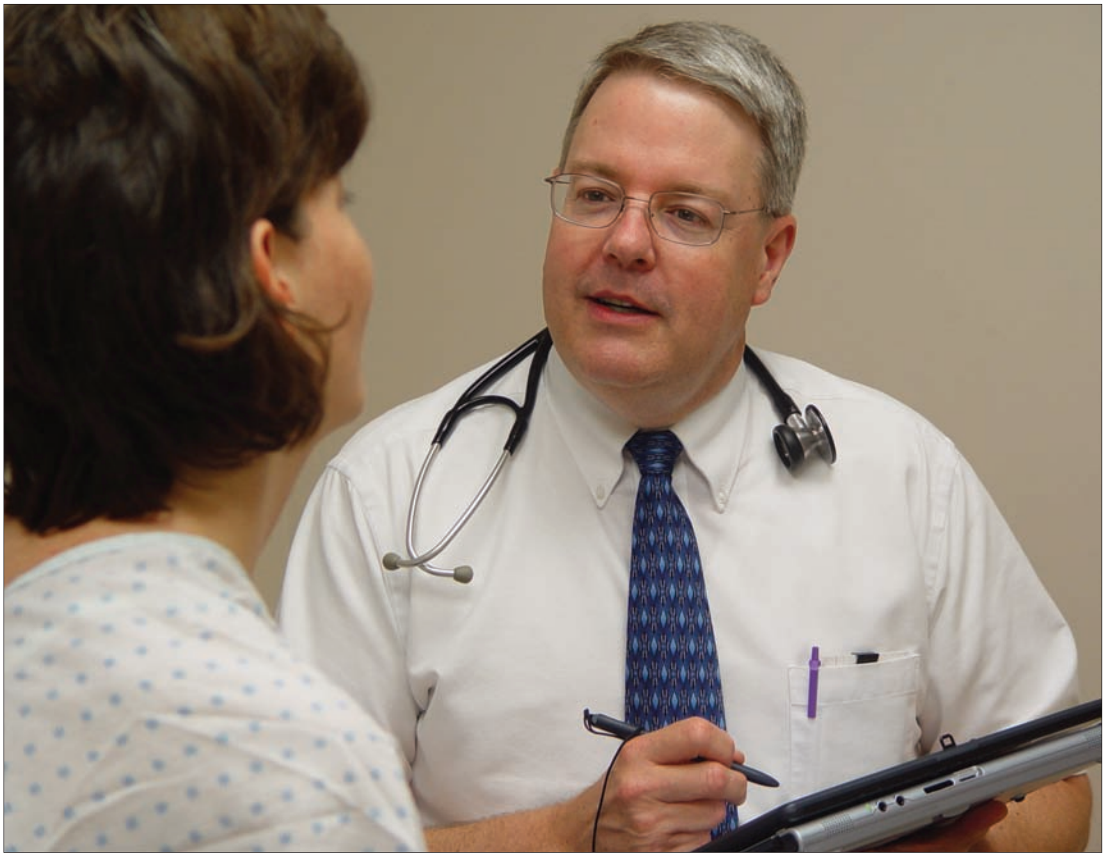

- What are electronic health records?
- How can electronic health records help?
- Who will have access to my electronic record?
- How will my information be protected?
- Is the *eHealth Summary* right for me?

This booklet is published by the Northern Berkshire *eHealth* Steering Committee. This committee represents many health care providers who are working together on a pilot project to develop a community-wide electronic health network. The goal of this project is to improve health care quality, cost, and safety.

Steering Committee members include:

David Delano
Director of Information Technology, Northern Berkshire Healthcare

Ronald B. Durning, Jr., MD
Hospitalist, North Adams Regional Hospital

Tina Gallant
Director of Support Services, Williamstown Medical Associates

Robert Jandl, MD
President, Williamstown Medical Associates
Chair, Northern Berkshire Healthcare Ethics Committee

Douglas Karrel, MD
Internist, Adams Internists

Richard Palmisano, R.N., M.S.
President & CEO, Northern Berkshire Healthcare

Anthony Smeglin, MD
Pulmonologist, Williamstown Medical Associates Physician Advocate, Northern Berkshire eHealth Collaborative

Stephen St. Clair , MD
Urologist, Associates in Urology

Holly Taylor, RHU
Chair, eHealth Community Committee
Vice President, True North Financial Services

William White
Executive Director, Williamstown Medical Associates

"There is no one in medicine who does not consider it both crucial and long overdue to have electronic records in doctors' offices and hospitals."

- New York Times on June 21, 2005

This pilot project is funded by the Massachusetts eHealth Collaborative (MAeHC). Northern Berkshire County is one of three communities selected by MAeHC in 2005 to demonstrate the use of electronic health record systems in doctors' offices. This project has received nationwide praise as a major advance in health care.

NBeHC
Northern Berkshire eHealth Collaborative
71 Hospital Avenue • North Adams, MA 01247 • (413) 664-5000 • www.nbehealth.com

A Message from Your Doctor

We are taking part in an exciting program to improve your health care and make office visits easier and more convenient. Beginning in late 2006, most doctors' offices in Northern Berkshire will begin using electronic (computer) health records. These electronic health records will replace the paper charts we now use.

We believe that these electronic health records will allow us to better serve you, our patients. These records will have a lot of important and up-to-date health information which we can use to provide high quality care.

This booklet describes two kinds of electronic health records. It includes answers to these important questions:

- What are electronic health records?
- How can electronic health records help?
- Who will have access to my electronic health record?
- How will my information be protected?
- Is the *eHealth Summary* right for me?

We hope that you will share our excitement about the many benefits of electronic health records. This booklet is a great place to start learning the facts. You can also learn more by talking with staff at your doctor's office and going to the eHealth website at www.nbehealth.com

Thank you for joining us in this effort!

What are electronic health records?

Electronic health records are a way for your physician to record and store health information on a computer. In the past, your doctor kept a paper chart. Now, with electronic health records, a doctor or other healthcare provider (nurse, physician assistant or nurse practitioner) will enter your health information into a secure (safe) computer record. This means that, with your permission, a doctor may share your important health information with other doctors in the community and coordinate your care.

Here are some facts about your *eHealth Record* and an *eHealth Summary*:

eHealth Record

This will simply be a computer version of your paper medical chart. It includes information about illnesses, family history, prescriptions, test results, and your health history. Your *eHealth Record* replaces the paper chart.

eHealth Summary

The *eHealth Summary* is a way your doctor can use a computer to share certain information from your *eHealth Record* with other providers. Until now, your doctor's office shared this information by mail, fax, or phone. This could take a lot of time and sometimes doctors did not get all the information they needed. The *eHealth Summary* will make this process faster and easier. For instance, if you have an *eHealth Summary,* then doctors at North Adams Regional Hospital can see your chart if you are admitted to the emergency room.

Your *eHealth Summary* will only show your medications, allergies, current medical problems, recent test results and other related summary information. It does **NOT** include all the details from medical exams or your doctor visits.

Your *eHealth Summary* may also include information about medical conditions that are considered "sensitive" under Massachusetts General Law. This can be about mental health or infectious and venereal diseases. Information about your HIV/AIDS status and any genetic testing will **NOT** be added to the *eHealth Summary* without your permission. To learn more about the types of information considered "sensitive" by the Commonwealth of Massachusetts, go to www.nbehealth.com.

You will have an *eHealth Record* at each of your doctor's offices, just as you have a paper chart at each office today.

And like your paper chart, your *eHealth Record* at each of your doctor's offices will only be available to that doctor's office.

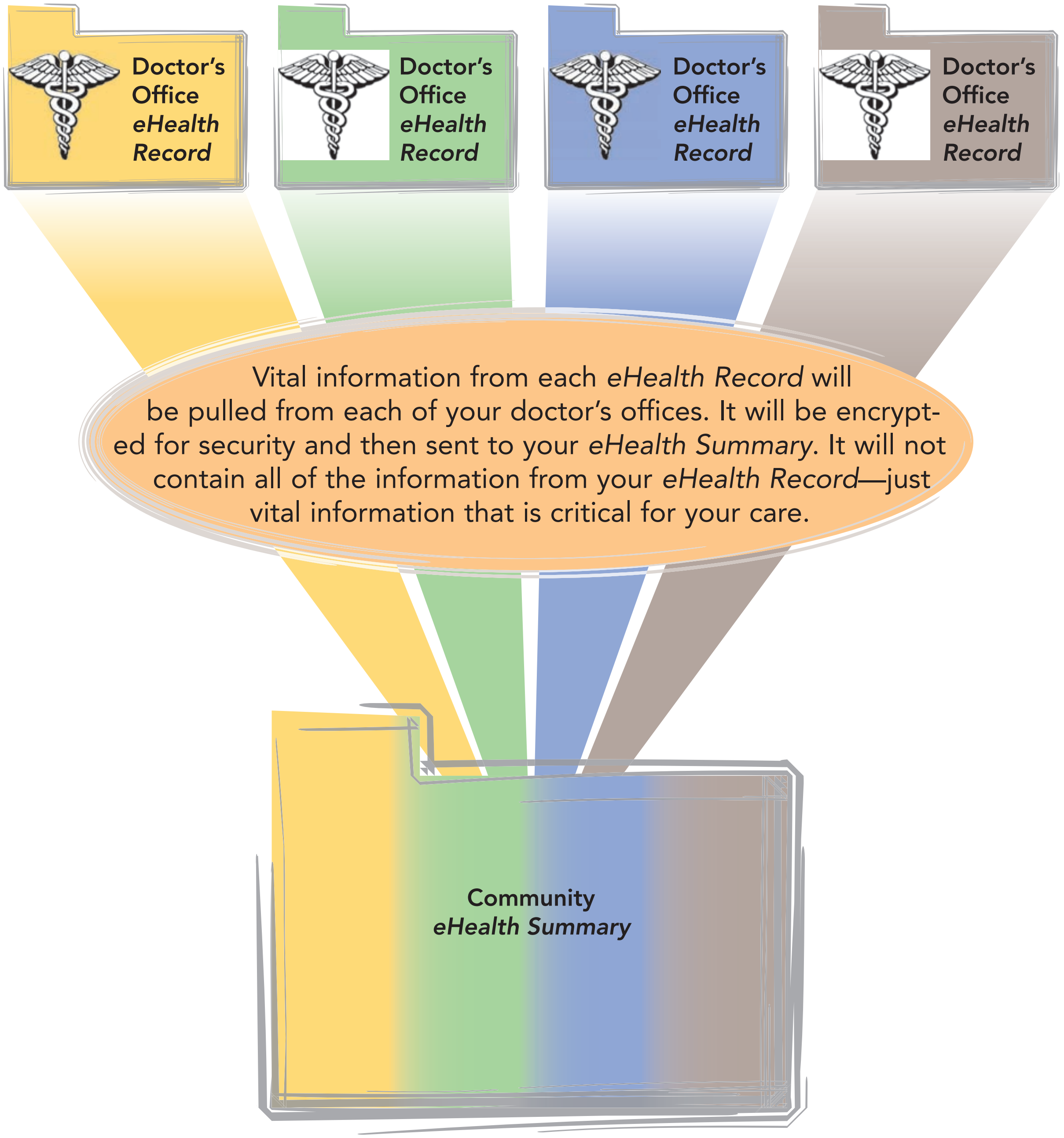

Your *eHealth Summary* will be put together from vital information from each *eHealth Record*.

This summary of your vital medical information will be available to doctor's offices and to the hospital, including the Emergency Department.

How can electronic health records help?

Your *eHealth Record* will:

- **Give your doctor automatic health reminders.** These include alerts if you need a colonoscopy, pap smear, mammogram, or other routine screening test.
- **Save time.** Once your *eHealth Record* is active, you and your doctor can spend less time filling out paperwork (like health histories and insurance forms) and more time talking about your treatment and care.
- **Improve medication recalls.** Prescription medications are sometimes recalled for safety reasons (meaning that you need to stop taking them). If this happens with a drug you are taking, your doctor can quickly let you know what to do. This is much harder to communicate with the paper charts that are now used.
- **Provide back-up in case of disaster.** In case of a fire or other disaster, your doctor will likely be able to save your important health information. Recent disasters like Hurricane Katrina (in which hundreds of thousands of people were left without medical records) show how important this can be.

If you give permission for an *eHealth Summary,* it will allow your doctors to:

- **Work better as a team.** The *eHealth Summary* can be shared by all the providers who treat you. This will help them work more as a team because they all can access the same information. Also, your doctors will get automatic updates about your health.
- **Quickly find information in an emergency.** When you have an *eHealth Summary*, authorized providers will have fast access to your health summary. They can quickly find information about your medications, allergies, health problems, and health care proxy. Finding this information quickly may save your life in an emergency.
- **Avoid drug interactions.** Your *eHealth Summary* can help reduce the risk of dangerous drug interactions (when one drug should not be mixed with others). This is a common and very serious problem. For instance, people sometimes die if they take certain heart medications along with common prescriptions like Viagra.

How the features of Paper Medical Charts and Electronic Health Records compare

A ✔ means that feature is available . . .

Features	Paper Medical Records	Electronic Health Records
Patients have the right to request a copy of their medical records.	✔	✔
Staff at the doctor's office is trained to protect the privacy and security of your records.	✔	✔
There is a way to keep track of who looks at your record, what section they look at, and when they looked at it. Patients can request this information.		✔
There is a way to keep track of new information. This includes the date, time, and name of anyone who adds, removes, or changes your health information.		✔
Records are protected by technology, which includes firewalls, intrusion detection, passwords and secure technology.		✔
There is more than one level of access to information. For instance, doctors can access more sensitive or personal information than can some office staff.		✔
Doctors can quickly and easily find a patient's important medical information - even in an emergency.		✔
Important health information is not likely to be lost in case of a fire or other disaster.		✔
There are automatic "alarms" if someone looks at a chart but is not authorized to do so. Doctors can also add new rules about who can access charts. Anyone who breaks these rules will be punished.		✔

Who will have access to my electronic record?

Electronic health records have strict rules about who can access your records and what information they can look at. These rules include:

- Office staff will only be able to access the information they need to do their job. For instance, front desk staff may only be able to view general information such as your name, address, and health insurance information. Your nurse or doctor can access additional information about your illnesses, medications, health history, and test results.
- If you have an *eHealth Summary*, everyone who can access this record must be trained and certified in privacy protection.
- As a patient who has an *eHealth Summary*, you have a right to see a list of all authorized providers who access your records. You also have a right to receive a copy of the privacy and security policies and guidelines. To learn more, you can ask staff at your doctor's office or go to the www.nbehealth.com website.
- You have the right to request from your doctor's office a full listing of everyone who has accessed your *eHealth Record* or your *eHealth Summary*.

"People rarely plan to come to the emergency room. As a result, they often don't have key information about their health that a doctor needs to effectively treat them. The electronic health record makes this critical information available in emergencies - easily, safely, and securely."

- Dr. Paul Donovan, Director, Emergency Room, North Adams Regional Hospital

How will my information be protected?

Your health information is protected by many layers of security. These include strict rules about who can access your information and what information they can read or share. There is also computer technology that keeps track of who accesses your record and when. If any of these rules are ever broken, there are harsh penalties for the people involved. Here is more information about these layers of security:

- **Technology.** Both your *eHealth Record* and your *eHealth Summary* are protected by advanced technology that is as good or in many ways better than the technology that banks use to protect financial data. This technology includes: firewalls, intrusion detection, secure communications (SSL), password protection, physical hardware security, automatic alarms for suspicious activity, and task-based access (so that non-clinical staff can be "blocked" from viewing medical information). The *eHealth Summary* is also protected by data encryption, so that even if a non-authorized user were able to break through all of these security walls, the medical information would not be readable.
- **Monitoring.** Your *eHealth Record* and *eHealth Summary* will also track everyone who has accessed any part of your electronic health records. This includes a list of people who have looked at your medical record, when they looked at it, and what changes (if any) they made. You can request a copy of this list from your doctor's office. Also, your doctor's office and the Steering Committee members listed in the front of this booklet will use this list to check for any user violations (if someone without authorization looks at your record). This type of computer monitoring is much more advanced and secure than with today's paper charts.
- **Alerts.** If anyone tries to access your information without authorization, the computer will send out an automatic alert to your doctor's office and the Steering Committee.
- **Audits.** Doctors' offices and the hospital must perform regular audits (checks) to make sure that no one is accessing medical information inappropriately.

(continued . . .)

Penalties

Your doctor's office and the Steering Committee, along with the state and federal government, are working to make sure that the rules of electronic medical records are followed. Here are some ways:

- **Laws.** It is a crime for anyone who is not directly involved with your health care to look at or disclose your medical information without your permission. Federal and state laws strictly regulate such violations. Failure to obey these laws can result in fines as high as $250,000 and prison terms of up to 10 years.

- **Sanctions.** Your doctor's office and the eHealth Steering Committee will investigate any reports of violations to the privacy policy. Unintentional violations (where it is determined that the violator did not follow the policy by accident) will result in penalties ranging from warning letters to permanent suspension of access to medical records. Intentional violations (when someone deliberately accesses a patient's record for reasons that aren't related to your care) will result in suspension of access, and prosecution to the fullest extent of the law.

- **Notifications.** Under certain circumstances your doctor's office will notify you if there is a privacy violation to your records. Please ask your doctor's office or see www.nbehealth.com for more information about the Notification of Security Violation policy.

Are there privacy risks?

Yes, as with any computerized system, there are some risks although reasonable precautions have been taken.

- The risks include the possibility that your medical history could indicate specific conditions such as sexually transmitted diseases, mental health, pregnancy, HIV status, genetic conditions, chronic conditions and alcohol or drug problems, or other conditions that you may consider sensitive. Other risks may include unauthorized individuals gaining access to the computer network, an authorized user misusing the health information and an authorized person seeing information in your record that makes you uncomfortable.

- There is also the possibility of inaccurate information being accidentally entered into your electronic record and shared with others through your eHealth Summary which could have a negative impact on your care.

Is the *eHealth Summary* right for me?

Your doctor needs your permission to start an *eHealth* Summary for you. Before you sign permission, here are some questions to think about. If you answer "yes" to any of them, then an *eHealth Summary* is likely right for you.

yes___ no___ I regularly take medications (prescription or over the counter).

yes___ no___ I am treated by two or more doctors

yes___ no___ I sometimes get treatment at the North Adams Regional Hospital.

yes___ no___ I have food, medication, or other types of allergies.

yes___ no___ I don't like filling out a lot of paperwork when I go to the doctor or hospital.

yes___ no___ I want all my doctors to have the most up-to-date medical information about me.

yes___ no___ In case of an emergency, I want the doctors to know about my health history, medications, and allergies.

Is an *eHealth Summary* right for you? If you agree with us that it is, then please sign the consent form at your doctor's office.

Ways to Learn More

- Talk with your doctor or the staff at your doctor's office.
- Go to www.nbehealth.com for more detailed information.
- Health Care for All consumer ehealth site: http://www.hcfama.org
- Connecting Communities for Better Health: http://www.ehealthinitiative.org
- Office of the National Coordinator for Health IT: http://www.hhs.gov/healthit

NBeHC
Northern Berkshire eHealth Collaborative
71 Hospital Avenue • North Adams, MA 01247 • (413) 664-5000 • www.nbehealth.com
