# Supplementary material for: Patients’ Attitudes Toward Electronic Health Information Exchange: Qualitative Study
Source: J Med Internet Res. 2009 Aug 6;11(3):e30. doi: 10.2196/jmir.1164 (PMC2762851; doi:10.2196/jmir.1164)
Supplement: Supplementary file 5 [file jmir_v11i3e30_app5.pdf]

NBeHC

Northern Berkshire eHealth Collaborative

— Important Information on Electronic Health Records —

To the Northern Berkshire Community:

We are taking part in an exciting program to improve your health care and make office visits easier and more convenient. Over the next several months, most doctors' offices in Northern Berkshire will begin using electronic (computer) health records. These electronic health records will replace the paper charts we now use.

We believe that these electronic health records will allow us to better serve you, our patients. These records will have a lot of important and up-to-date health information which we can use to provide the highest quality care.

What is an electronic health record?

An electronic health record is a way for your physician to record and store health information on a computer. In the past, your doctor kept a paper chart. Now, with electronic health records, a doctor or other healthcare provider (nurse, physician assistant or nurse practitioner) will enter your health information into a secure computer record, or *eHealth Record*. This means that, with your permission, a doctor may share your important health information with other doctors in the community.

Your doctors will now have your most important medical information at their fingertips. They will receive automatic reminders and alerts about your preventive health needs. If one of your prescriptions is recalled, your doctor can quickly let you know what to do. And in case of a disaster, such as a fire, it is more likely that your important health information will be saved.

You may also sign up for an *eHealth Summary*, which makes it easier for your doctor to share certain information with other providers. Until now, your doctor's office shared this information by mail, fax, or phone. But this could take a lot of time and sometimes doctors did not get all the information they need. The *eHealth Summary* will make this process faster and easier.

This summary will only show your medications, allergies, current medical problems, and recent test results. It does NOT include all the details from medical exams or doctor's visits.

Who is participating?

A list of participating physician practices is provided on the reverse side.

What do I need to do?

Please be 10-15 minutes early for your next appointment if you are seeing a participating doctor. You will need to read the paperwork to sign up for the *eHealth Summary*.

Please know that your doctor's office is undergoing a major change – one that they have adopted because it is best for your care. If things run a little slower than usual, please be patient. Your doctor's office may still be getting used to this new system.

We know how important your health care is, and we are pleased to offer this technology to you.

77 Hospital Avenue • North Adams, MA 01247 • (413) 664-5000 • www.nbehealth.com

How will my information be protected?

Your health information will be protected by many layers of security. These include strict rules about who can access your information and what information they can read or share. There is also computer technology that keeps track of who, what, and when people are accessing your records. If any of these rules are ever broken, there are harsh penalties for the people involved. You may request a list of who has viewed your record and when. Regular audits will be done to ensure that no one has improperly accessed your record. Each practice is fully committed to the privacy, security, and confidentiality of your medical record.

The following practices will be adopting electronic medical records over the upcoming weeks and months:

- Associates in Urology
- Adams Internists
- Berkshire Hematology Oncology
- Greylock Gastroenterology
- Herr, Douglas V, MD
- Hertzig, Gerrity, Griffin, & Degrenier
- Howland, John MD
- Integrative Medicine
- Kratzer, Joseph MD
- Maher, Paul MD
- North Adams Regional Hospital
- Northern Berkshire Family Practice
- Northern Berkshire Sports Medicine
- Orthopedic Associates of Northern Berkshire
- Sweet Brook Transitional Care and Living Centers
- Tanzman, Maselli, and Associates
- Van Uitert, Robert L. MD
- VNA + Hospice of Northern Berkshire
- Williamstown Medical Associates
- Yurfest, Joshua T. M.D.

With your permission, North Adams Regional Hospital, the VNA + Hospice of Northern Berkshire, and Sweet Brook Transitional Care and Living Centers may have access to an *eHealth Summary*. Your consent is required before an *eHealth Summary* is created for you.

This list may change over time, as new practices are added and deleted, and at any time, you may request a list of current participants from your doctor's office.
